# On the error propagation of semi-Lagrange and Fourier methods for advection problems^☆^

**DOI:** 10.1016/j.camwa.2014.12.004

**Published:** 2015-02

**Authors:** Lukas Einkemmer, Alexander Ostermann

**Affiliations:** Department of Mathematics, University of Innsbruck, Austria

**Keywords:** Semi-Lagrange methods, FFT, Error propagation, High-precision computations

## Abstract

In this paper we study the error propagation of numerical schemes for the advection equation in the case where high precision is desired. The numerical methods considered are based on the fast Fourier transform, polynomial interpolation (semi-Lagrangian methods using a Lagrange or spline interpolation), and a discontinuous Galerkin semi-Lagrangian approach (which is conservative and has to store more than a single value per cell).

We demonstrate, by carrying out numerical experiments, that the worst case error estimates given in the literature provide a good explanation for the error propagation of the interpolation-based semi-Lagrangian methods. For the discontinuous Galerkin semi-Lagrangian method, however, we find that the characteristic property of semi-Lagrangian error estimates (namely the fact that the error increases proportionally to the number of time steps) is not observed. We provide an explanation for this behavior and conduct numerical simulations that corroborate the different qualitative features of the error in the two respective types of semi-Lagrangian methods.

The method based on the fast Fourier transform is exact but, due to round-off errors, susceptible to a linear increase of the error in the number of time steps. We show how to modify the Cooley–Tukey algorithm in order to obtain an error growth that is proportional to the square root of the number of time steps.

Finally, we show, for a simple model, that our conclusions hold true if the advection solver is used as part of a splitting scheme.

## 1 Introduction

The accurate numerical simulation of advection dominated problems is an important problem in many scientific applications. However, due to the non-dissipative nature of the equations considered, care has to be taken to obtain a stable numerical scheme (see, for example,  [1]). A large body of research has been accumulated that describes finite difference, finite volume, and finite element discretizations of such problems. However, for advection-dominated problems so-called semi-Lagrangian methods offer a competitive alternative. These methods integrate the characteristics back in time and consequently have to use some interpolation scheme to reconstruct the desired value at the grid points. Strictly speaking, semi-Lagrangian methods can only be applied to systems of first-order differential equations. However, in many instances, first-order systems arise from the splitting of more complicated equations or constitute the linear part of an evolution equation (which is then treated separately from the nonlinearity). Consequently, semi-Lagrangian methods have been used extensively in applications ranging from fluid dynamics to plasma physics (see e.g.  [2,3]). Such an approach is especially promising, if the characteristics (of a sub-problem) can be computed analytically; this can be done, for example, in context of the Vlasov–Poisson equations. In addition, semi-Lagrangian methods do not impose a Courant–Friedrichs–Lewy (CFL) condition.

In some problems, methods based on the FFT (fast Fourier transform) can also be employed; this is the case for tensor product domains (see e.g.  [4]). Compared to FFT based methods, the semi-Lagrangian methods provide a local approximation (which is important in the context of parallelization). They are more easily applicable to non-periodic boundary conditions (due to the absence of Gibbs’ phenomenon) and usually are better suited to handle nonlinearities. Using the fast Fourier transform, on the other hand, allows us to solve the linear advection equation exactly (in infinite precision arithmetics).

In most scientific applications a tolerance of say 10^−3^ is sufficient. In this case, the main research goal is to construct more efficient algorithms and to implement better step size control mechanism. Also methods that preserve certain invariants of the continuous system are of interest in that context.

However, a number of applications have been identified where double precision floating point numbers are not sufficient. A proposed remedy is to (selectively) use 128-bit floating point numbers. Note, however, that this procedure is accompanied by a significant reduction in performance. Examples of such problems range from the investigation of vortex sheet roll-ups in fluid dynamics to electromagnetic scattering phenomena (for an excellent review article see  [5]).

Also, concern has been raised in recent years with regard to the reproducibility of numerical simulations; especially if such simulations are conducted on different computer systems. In  [6], for example, it is demonstrated that climate codes show significantly different results depending on the number of processors that are employed in the simulation. One popular choice of numerical methods for atmospheric modeling are semi-Lagrangian methods.

Furthermore, due to the diminishing gain in per core CPU (central processing unit) performance, massively parallel computing architectures, such as GPUs and the Xeon Phi, have become more and more common. Usually in such situations the memory per core is significantly smaller than in more traditional cluster systems. In addition, single precision floating point performance is usually faster than double precision floating point performance (for example, the CUDA FFT single precision implementation achieves a speedup of about 2.5 compared to the double precision implementation  [7]). Such considerations make the use of single precision floating point numbers attractive for some applications.

Therefore, our goal in this paper is to study the error propagation in a context where results close to machine precision are of interest or where the error has to be tightly controlled. We will limit ourselves to the advection equation (on a finite spatial interval)

(1) $$\partial_{t}u\left(t,x\right)+v\partial_{x}u\left(t,x\right)=0\text{,}$$

where u is a continuously differentiable function and v is a given constant.

For this model problem, we consider the time evolution of the interplay of round-off and discretization errors for semi-Lagrangian and FFT based methods. The discretization of (1) is important in itself as it is a building block for many more involved schemes (for example, in the context of splitting methods or for methods where the linear part is treated differently). Such schemes are applied, for example, in fluid dynamics or to solve the Vlasov equation. Note, however, that problems where v is a function of space and time can be treated as well within the semi-Lagrangian approach. In this case the characteristics have to be integrated backward in time by a suitable ordinary differential equation solver. In certain situations a function v which depends on the unknown function u can be handled as well (see, for example,  [8]).

For future reference, we note that the analytic solution of (1) can be easily written down as

u(t,x)=u(0,x−vt),

where in this paper we always assume that v is a constant independent of x and t.

## 2 Description and error bounds

In this section we will describe how to use the semi-Lagrangian method (Section  2.1) and the fast Fourier transform (Section  2.2). Furthermore, we will discuss some theoretical results concerning the discretization error of these schemes.

### 2.1 The semi-Lagrangian method

A time step in the semi-Lagrangian method for the ith grid point is computed as follows:

$$u_{n}\left(x_{i}\right)=u_{n-1}\left(X_{x_{i}}\left(\tau \right)\right)\text{,}$$

where $u_{n}$ is the numerical solution after n time steps, τ is the time step size, and the characteristics of Eq. (1) are given by $X_{x}\left(\tau \right)=x-v\tau$. Note that $X_{x_{i}}\left(\tau \right)$ does not necessarily coincide with any grid point. Therefore, a sufficiently accurate interpolation procedure has to be used in order to extend the values stored at the grid to the entire domain. Both (continuous) spline interpolation as well as (discontinuous) Legendre or Lagrange interpolation are popular choices. Furthermore, similar to discontinuous Galerkin methods, multiple coefficients can be stored for each cell (which yields a local reconstruction at the expense of additional memory demands).

It is well known (see e.g.  [9,10]) that in the case of semi-Lagrangian methods the error of the fully discretized problem can be estimated by

(2) $$\left\|u\left(n\tau ,x\right)-u_{n}\left(x\right)\right\|\leq C\left(h^{q}+\frac{h^{q}}{\tau }\right)\text{,}$$

where h is the grid size, and q is the order of the space discretization (for example, the order of the Lagrange interpolation). Note that C is a constant that is independent of n, τ, and h. Such estimates are usually derived in one (or all) of the $L^{p}$ norms. Note that there is no term proportional to τ as the characteristics are known analytically. However, results can be derived that take time discretization errors into account (see e.g.  [9,10]).

The first term on the right-hand side in Eq. (2) is the usual spatial error term. The disturbing implication, however, is that the second term is directly proportional to the number of time steps taken. This, however, is to be expected, as in each projection an error proportional to $h^{q}$ is made. In the worst case these errors accumulate to give the above mentioned bound. Note that the second term is usually not present if Eulerian discretization methods (for example finite difference schemes) are employed.

Similarly how the discontinuous Galerkin (dG) method considers a polynomial reconstruction that only uses data attached to the corresponding cell, it is possible to formulate a semi-Lagrangian method based on this reconstruction. This dG semi-Lagrangian method also requires only the data from neighboring cells in order to perform a time step (for arbitrary order in space). The scheme is described in some detail in  [11,10]. Since there is no ambiguity, in this paper, we will refer to this scheme as the dG approximation.

Let us duly note that the second term in Eq. (2) is a worst case estimate that is valid for all the interpolation and projection schemes discussed here. Thus, in the next section we will investigate the actual numerical behavior for the spline interpolation, the Lagrange interpolation, and the discontinuous Galerkin approximation.

### 2.2 The fast Fourier transform

In case of the FFT based scheme we compute the Fourier transform of (1) which gives

(3) $$\partial_{t}\overset{ˆ}{u}\left(t,k\right)+ikv\overset{ˆ}{u}\left(t,k\right)=0\text{,}$$

where we have denoted the Fourier component with frequency k∈Z by $\overset{ˆ}{u}\left(t,k\right)$. We can easily solve (3) to get

$$\overset{ˆ}{u}\left(t,k\right)=e^{-ikvt}\overset{ˆ}{u}\left(0,k\right)\text{.}$$

Therefore, the advection in Fourier space is described by the multiplication with an appropriate phase factor. The numerical scheme truncates the Fourier series. Therefore, we only consider −m≤k≤m. Note that in the numerical implementation we employ the so-called discrete Fourier transform (DFT). The DFT replaces the integral necessary to determine the Fourier coefficient by a quadrature formula. It can alternatively be interpreted as a (trigonometric) polynomial interpolation.

Since u(t,k) is a real function, only the non-negative frequencies have to be stored in memory. If exact arithmetics is used, we obtain an error bound that depends on the spatial regularity of the solution. For $u\left(t,\cdot \right)\in C^{q}\left(a,b\right)$, with a<b, and u(t,⋅) periodic in the first q derivatives we have

$$\left\|u\left(n\tau ,x\right)-u_{n}\left(x\right)\right\|\leq Ch^{q-1}\text{,}$$

where h=(b−a)/m is the grid size. In particular, for periodic $u\left(t,\cdot \right)\in C^{\infty }\left(a,b\right)$ the convergence is super-polynomial in the number of grid points. This is the only discretization error provided that exact arithmetics is employed.

## 3 Numerical investigation

The purpose of this section is to present the results from a number of numerical simulations conducted. It will soon be apparent that the actual computations display a more complicated behavior as would be expected from the error estimates discussed in the previous section. Note that in all the numerical simulations conducted we have chosen to discretize problem  (1) using v=1.

**Fig. 1:**
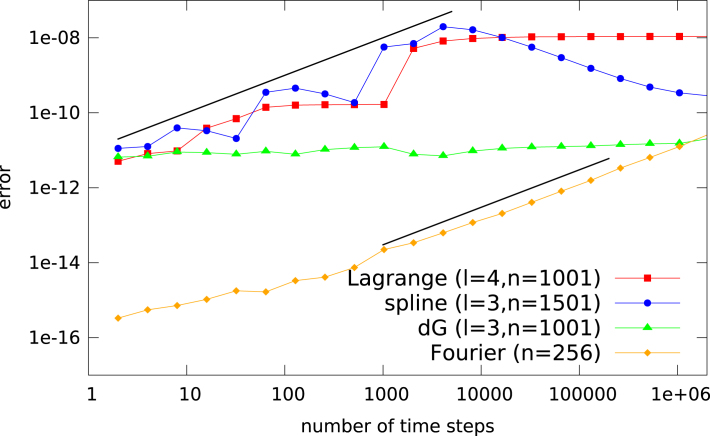
The $L^{\infty }$ error of the Lagrange, spline, dG, and FFT based methods, as a function of the number of time steps, is shown. The FFT routine from the FFTW library is used. The polynomial degree is denoted by l and the number of cells/grid points is denoted by n. As a reference two black lines of slope 1 are drawn.

In Fig. 1, we compare the error propagation for a Lagrange interpolation, a discontinuous Galerkin method, and the Fourier approximation using the initial value

(4) $$u\left(0,x\right)=\frac{1}{2+\cos\pi x}$$

on the interval [−1,1] with periodic boundary conditions (the same interval and periodic boundary conditions are used for all simulations in this paper). As expected, initially the FFT method achieves a performance close to machine precision. Note, however, that the error growth is linear in the number of time steps. However, from a stochastic description of the round-off error one would expect an error growth proportional to the square root in the number of time steps. Let us postpone the detailed investigation of this issue until Section  5.

Furthermore, even though the worst case error estimate for a general semi-Lagrangian method does include the term proportional to the number of steps, this is only observed for the Lagrange and the spline interpolation (see Fig. 1). However, the dG method does not exhibit such a behavior. In fact, there is almost no error propagation even after more than 10^6^ steps in time have been conduced. In this instance, an error in the initial value that is orders of magnitude away from machine precision can still be competitive with the FFT based scheme (which shows a linear propagation of the error).

For both the Lagrange and the spline interpolation some oscillations do occur. In general, however, a linear error propagation is observed.

**Fig. 2:**
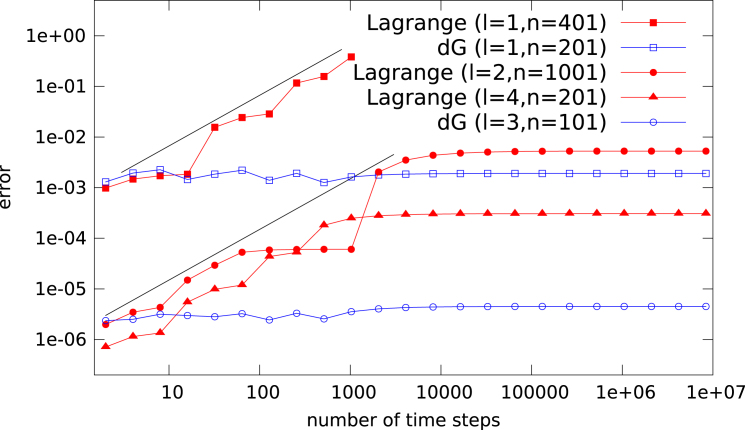
The $L^{\infty }$ error as a function of the number of time steps is shown for a number of different configurations (the interpolation degree l and the number of grid points/cells n of the Lagrange and dG interpolation methods are varied). The initial value given in (5) is used. As a reference two black lines of slope 1 are shown.

In Fig. 2 we compare different polynomial degrees for both the Lagrange and dG based methods with the initial value

(5) u(0,x)=cos4πx.

As before, we initially observe a linear error growth, which yields a reduction in accuracy of at least three orders of magnitude for the Lagrange interpolation. No such behavior is observed for the dG method.

## 4 A theoretical investigation of the semi-Lagrangian methods

In the previous section we observed a remarkable difference in the error propagation for the semi-Lagrangian methods employing Lagrange or spline interpolation on the one hand and the discontinuous Galerkin method on the other hand. The conjecture is that in case of the discontinuous Galerkin scheme the errors made in each step average to zero over the course of many time steps, while for the interpolation methods they are in fact well described by the worst case estimate stated in the introduction.

It is our goal now to provide an explanation for this behavior in the case of the linear Lagrange interpolation. For simplicity, let us choose v=1 in Eq. (1). In addition, in this section, we employ a notation which drops the time dependence of u. Thus, we denote the initial value for a given time step by u(x) and the ith grid point by $x_{i}$. A single time step of size τ (and with v=1) then gives the new value at the ith grid point (which we denote by $\overset{˜}{u}\left(x_{i}\right)$)

(6) $$\overset{˜}{u}\left(x_{i}\right)=\frac{1}{h}\left(\tau u\left(x_{i-1}\right)+\left(h-\tau \right)u\left(x_{i}\right)\right)\text{.}$$

Note that we have restricted ourselves to τ<h. This is justified as each advection with τ>h can be decomposed into an advection that can be solved exactly, where τ is a multiple of h, and an advection for which τ<h holds true.

The corresponding extension to the entire domain is then given by (where ξ∈[0,h] is restricted to the cell under consideration)

(7) $$\overset{˜}{u}\left(x_{i}+\xi \right)=\frac{1}{h}\left(\left(h-\xi \right)\overset{˜}{u}\left(x_{i}\right)+\xi \overset{˜}{u}\left(x_{i+1}\right)\right)\text{.}$$

Before we proceed, let us make two remarks. First, in what follows, we compute the error as compared to the advected initial value which lies in the space of piecewise linear functions (and not to the analytic initial value). This is in fact the correct choice as we are interested in the error propagation and not in the initial projection error (which clearly is bounded by $Ch^{2}$ in this case). Second, the error is a function of two variables; the position $x=x_{i}+\xi$ and the size of the time step τ.

Let us consider the average (with respect to the spatial variable in a single cell, which is denoted by ξ) for the exact advection

$$\frac{1}{h}\int_{0}^{h}u\left(x_{i}+\xi -\tau \right)\;d\xi =\frac{1}{h}\int_{h-\tau }^{h}u\left(x_{i-1}+\xi \right)\;d\xi +\frac{1}{h}\int_{0}^{h-\tau }u\left(x_{i}+\xi \right)\;d\xi =\frac{\tau^{2}}{2h^{2}}u\left(x_{i-1}\right)+\frac{{\left(h+\tau \right)}^{2}-3\tau^{2}}{2h^{2}}u\left(x_{i}\right)+\frac{{\left(h-\tau \right)}^{2}}{2h^{2}}u\left(x_{i+1}\right)$$

and for the linear Lagrangian interpolation (using (7) and (6))

$$\frac{1}{h}\int_{0}^{h}\overset{˜}{u}\left(x_{i}+\xi \right)\;d\xi =\frac{\tau }{2h}u\left(x_{i-1}\right)+\frac{1}{2}u\left(x_{i}\right)+\frac{h-\tau }{2h}u\left(x_{i+1}\right)\text{.}$$

Now, since we are interested in simulations where a large number of steps has to be conducted it is reasonable to assume that for a fixed cell in the computational grid the step size τ∈[0,h] is uniformly distributed across that interval. Thus, the average in a single cell (averaged in both space as well as step size) for the exact solution is given by

$$\frac{1}{h}\int_{0}^{h}\frac{1}{h}\int_{0}^{h}u\left(x_{i}+\xi -\tau \right)\;d\xi d\tau =\frac{1}{6}\left(u\left(x_{i-1}\right)+4u\left(x_{i}\right)+u\left(x_{i+1}\right)\right)\text{,}$$

whereas for the linear Lagrangian interpolation we get

$$\frac{1}{h}\int_{0}^{h}\frac{1}{h}\int_{0}^{h}\overset{˜}{u}\left(x_{i}+\xi \right)\;d\xi d\tau =\frac{1}{4}\left(u\left(x_{i-1}\right)+2u\left(x_{i}\right)+u\left(x_{i+1}\right)\right)\text{.}$$

The double averaged error $\bar{\bar{e}}$ in a single cell is therefore given by

$$\bar{\bar{e}}=\frac{1}{12}\left(u\left(x_{i-1}\right)-2u\left(x_{i}\right)+u\left(x_{i+1}\right)\right)\approx \frac{h^{2}}{12}u^{\prime \prime }\left(x_{i}\right)\text{.}$$

Since there is a non-zero average error this error is amplified in each time step and gives a linear error propagation. Similarly to this computation we can also derive results for higher degree Lagrange interpolation. For the case of piecewise quadratic polynomials, for example, we get

$$\bar{\bar{e}}=\frac{1}{144}\left(u\left(x_{i-2}\right)+2u\left(x_{i-1}\right)-12u\left(x_{i}\right)+14u\left(x_{i+1}\right)-5u\left(x_{i+2}\right)\right)\approx -\frac{h^{3}}{24}u^{\left(3\right)}\left(x_{i}\right)\text{.}$$

In contrast, the discontinuous Galerkin method by construction preserves the average exactly. This is a necessary condition (but not a sufficient one) in order for the errors to cancel out on average. This is, of course, a different way of stating that the dG scheme is locally conservative for any step size while the Lagrange interpolation is not. The fact that most semi-Lagrangian schemes are not conservative is well established in the literature. To remedy this deficiency usually high-order methods are employed which provide sufficient accuracy to keep the violation in mass conservation to an acceptable level.

However, in our context this is not a remedy since, as we have observed in the previous section, even a Lagrange interpolation that is of high accuracy will loose at least three orders of magnitude in precision after approximately 10^3^ time steps. This is no concern if an approximation correct to three digits is desired. However, it is a significant drawback if we are interested in accuracies close to machine precision.

Note that since the average error is proportional to the second derivative we would expect that this behavior can be observed in numerical simulations. To that end we consider the convex initial value

(8) u(0,x)=(x−1)(x+1)

and the concave initial value

(9) u(0,x)=−(x−1)(x+1).

**Fig. 3:**
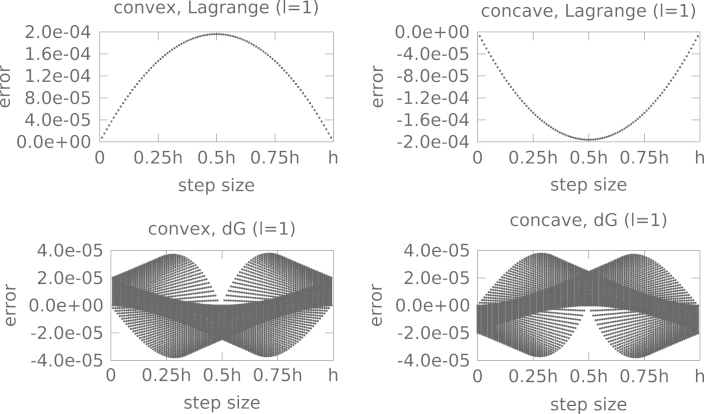
The error, after a single time step of size τ∈[0,h], where h is the cell size, is shown for the convex (8) and concave (9) initial values. In case of the Lagrangian interpolation the error at the different grid points is plotted at the same position on the x-axis (the value of which corresponds to the step size τ). Since the errors are almost identical they lie on a point. For the dG method the errors computed at different (equidistant) positions (within a single cell) are displayed at the corresponding τ value.

In fact Fig. 3 shows the expected behavior. Note that the errors made do depend on both the step size τ as well as the position x. For the Lagrange interpolation we have plotted the error at the different grid points at the same τ value (these points are almost identical and thus indistinguishable in the plot). In the case of the discontinuous Galerkin method we want to demonstrate that the error inside a single cell cancels out. Therefore, we have plotted the error at different points (but inside the same cell) at the same τ value.

**Fig. 4:**
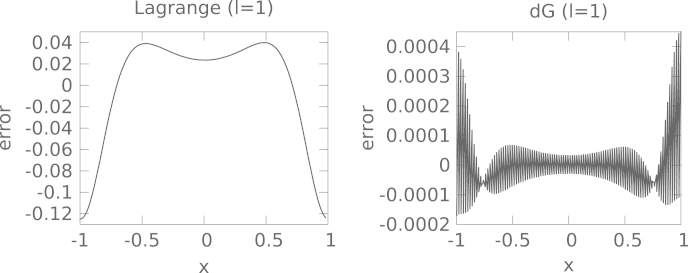
The pointwise error after 10^4^ steps of size $\tau =2\cdot 10^{-4}$ for the Lagrange and dG method (with 101 grid points) is shown. The left plot (Lagrange interpolation) matches the second derivative of (4) (the initial value used in this simulation). In case of the discontinuous Galerkin approximation the error displays small oscillations of high frequency.

In addition, we show the error as a function of the spatial variable in Fig. 4. As would be expected we observe an error that is similar to the second derivative of (4) for the Lagrange interpolation and an oscillatory error for the dG method.

## 5 Fast Fourier transform round-off error propagation

**Fig. 5:**
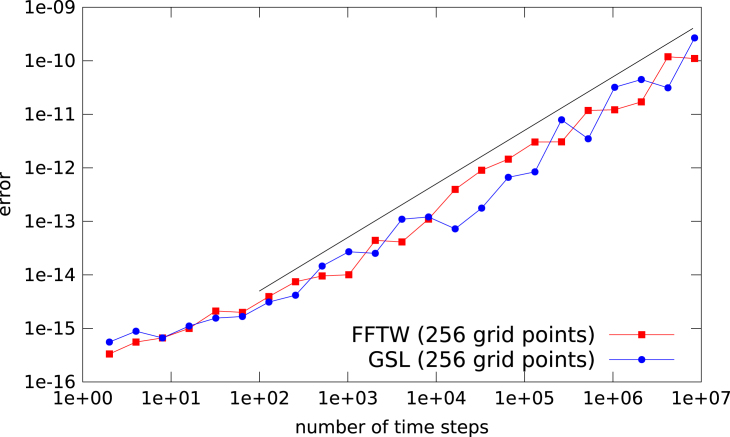
The $L^{\infty }$ error of the FFT based advection as a function of the number of time steps. Results for the FFTW and GSL library are shown. As a reference a black line with slope 1 is also displayed.

In the numerical simulations conducted in Section  3 we observed that the error growth is linear in the number of time steps (and not proportional to the square root as we would expect from a pure propagation of round-off errors). This behavior is consistent across numerical libraries; in Fig. 5 results using the FFTW (Fastest Fourier Transform in the West) library and the radix-2 implementation found in GSL (GNU Scientific Library) are shown.

**Fig. 6:**
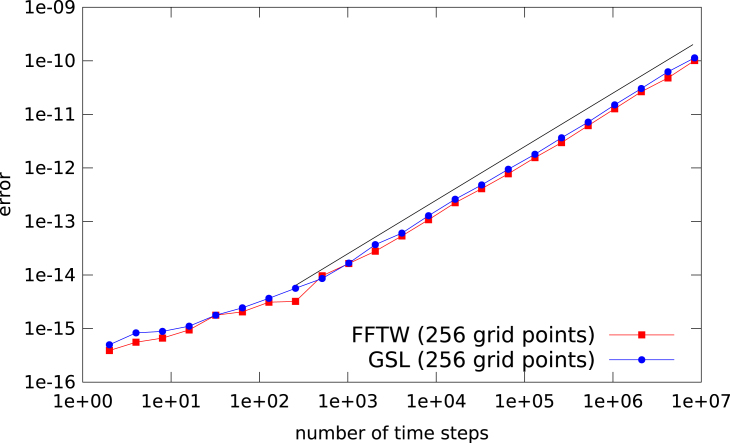
$L^{\infty }$ error of the FFT based advection as a function of the number of time steps. The phase factor needed for the translation and the corresponding combination with the signal is computed in high precision arithmetics and then rounded down to double precision. As a reference a black line with slope 1 is also shown.

An obvious explanation is to suggest that the phase factor used to compute the advection incurs some additional round-off errors. This phenomenon is well known in the context of reducing the round-off errors introduced by FFT routines (see e.g.  [12]). In that context, care has to be taken that the twiddle factors are computed to sufficient accuracy. A similar approach can be used to compute the phase factor in the advection. However, Fig. 6 clearly shows that the error growth is still linear in the number of time steps.

Note that the FFT algorithm as originally proposed by Cooley and Tukey is usually not implemented in high-performance FFT libraries (such as FFTW). A number of additional optimizations are performed. For the FFTW library a discussion can be found in  [13]. These optimizations often have a significant impact on accuracy. Therefore, accuracy benchmarks are performed in order to verify that the round-off errors are reasonable (see, for example,  [13]). However, in this paper we are interested in error propagation for a large number of time steps and not primarily with the scaling of the round-off error for large problem sizes.

**Fig. 7:**
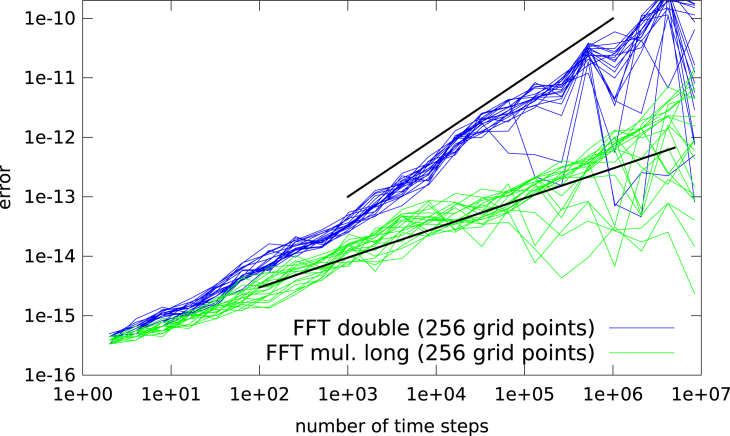
The $L^{\infty }$ error of the FFT based advection as a function of the number of time steps is shown for a number of different initial values as given in Eq. (10). The plain FFT implementation following the Cooley–Tukey algorithm (blue) and a plain FFT algorithm where the multiplications for the phase factor as well as in the Cooley–Tukey algorithm are carried out in 80-bit arithmetics (green) are compared. Note that in the latter case the storage requirement is still essentially the same (that is we do only have to store the input and output vector, in double precision, as well as the twiddle factors). As a reference two black lines with slope 1 (upper line) and slope 1/2 (lower line) are shown. (For interpretation of the references to color in this figure legend, the reader is referred to the web version of this article.)

In Fig. 7 the error propagation for the plain Cooley–Tukey algorithm is shown for the initial value

(10) $$u\left(0,x\right)=\frac{1}{2+\cos\left(\pi x+\varphi \right)}\text{,}$$

where φ is chosen at random. Even though the error propagation is significantly reduced compared to the FFTW implementation, at least four orders of magnitude are lost and the initial error growth is still linear.

If, in addition, the multiplications in the fast Fourier transform are computed to a higher precision, the error growth shows a behavior that is roughly proportional to the square root of the number of steps. In Fig. 7 the multiplications have been implemented in the 80 bit extended precision type^1^ implemented in the x86 hardware.

In the naive implementation used here we observe a reduction in performance of about 10%–15%. Note, however, that if vectorization is used such procedures can result in a more severe reduction in performance.

On the other hand, this approach would also be advantageous, if double precision floating point computations significantly impact performance (such as commonly found on GPU systems). In this case the input and the output would be stored as single precision floating point numbers and multiplications would be performed as double precision (with appropriately computed twiddle factors) and then correctly rounded to single precision.

## 6 Splitting of an advection equation with source term

**Fig. 8:**
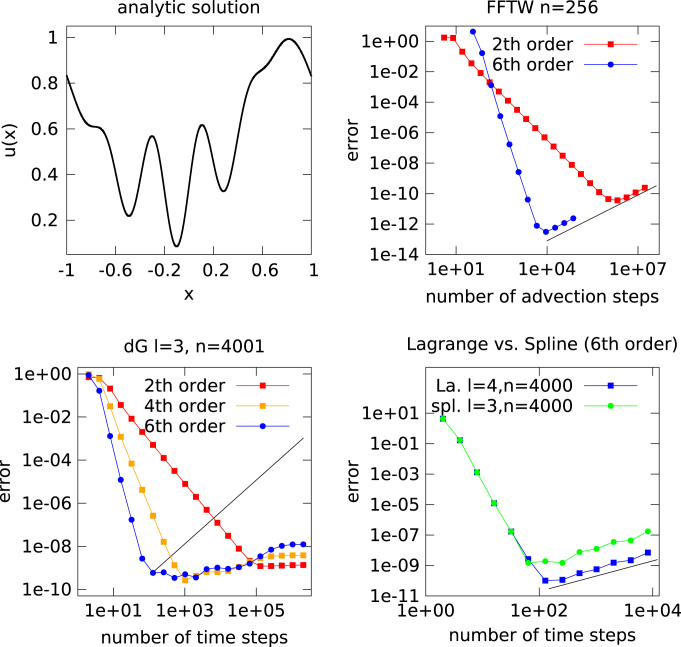
The solution of (11) is computed for the final time t=1.8. The analytical solution is shown on the top left. The numerical solution is computed using a splitting approach. The results for the FFT based method (top right), the discontinuous Galerkin (dG) method (bottom left), and the Lagrange and spline interpolations (bottom right) are shown. The black lines have slope 1 and are shown for comparison.

In this section we consider the advection equation supplemented by a (position dependent) source term. That is, we consider

(11) $$\partial_{t}u\left(t,x\right)+\partial_{x}u\left(t,x\right)=s\left(x\right)\text{,}$$

where the source term is chosen as s(x)=(1+cosπx)cos5πx and periodic boundary conditions are imposed. The solution can be easily determined by the method of characteristics

$$u\left(t,x\right)=u_{0}\left(x-t\right)+\int_{0}^{t}s\left(x-t+\sigma \right)\;d\sigma \text{,}$$

which for the initial value given in (4) is plotted in Fig. 8 (top left).

For the time integration we employ the second order Strang splitting scheme as well as the 6th order scheme constructed from it by composition (see, for example,  [14]). An approximation to the solution of the first sub-problem (the advection equation) can be computed as described in the previous sections. The remaining sub-problem (corresponding to the source term) is easily solved analytically. Note, however, that in the case of the dG method only the coefficients (in the Legendre expansion) are stored in memory. Thus, in each cell, we evaluate the approximation at the Gauss–Legendre points. We then compute the solution of the sub-problem on these points and use the result to reconstruct the coefficients.

The numerical simulations conducted confirm the observations made in the previous sections. For the FFT based method (using the FFTW library) we see, after a decrease in the error due to the time discretization error, the characteristic linear increase in the error (see Fig. 8 top right). Furthermore, we observe that, as expected, the lowest error we can achieve for a given time discretization is only dependent on the number of advection steps that have to be made in order to reach that tolerance (thus the method of order six is clearly the preferred choice in this case). All these phenomena are due to round-off errors only.

The spline and Lagrange interpolations show a similar behavior. However, in this case the linear increase in the error (for further decreasing step size) is dependent on the space discretization (see Fig. 8 bottom right).

For the dG method, on the other hand, the minimal error that can be achieved (for a given space discretization) is almost independent of the numerical method used in time. Of course, the 6th order method is usually more efficient if high precision is desired. However, if a large number of time steps are taken with the Strang splitting scheme, a similar accuracy than for the 6th order method can be achieved (without changing the space discretization). Furthermore, there is no linear increase in the error. All these observations are in line with the observations made in Sections  3 and 4.

## 7 Conclusion

In this paper we have considered the error propagation for the advection equation in the case where high precision is desired. The numerical methods considered exhibit a variety of different phenomena.

In case of the fast Fourier transform method round-off errors are the primary concern. A number of libraries that implement the FFT show a linear error growth in the number of time steps. However, if the multiplication of the Fourier coefficients with the twiddle factors is performed to sufficient accuracy the growth in the error is only proportional to the square root in the number of time steps.

Furthermore, we have shown that the term proportional to the number of time steps, that is routinely obtained in error estimates for semi-Lagrangian methods, is not observed for all semi-Lagrangian schemes. In fact it is true that the qualitative features of the error are markedly different for the interpolation (Lagrange as well as spline) and the discontinuous Galerkin based semi-Lagrangian schemes considered in this paper.

## Footnotes

1 This data type is usually mapped to long double by the C/C++ compiler. Note, however, that this is not required by the C/C++ standard and is not entirely consistent across different compiler implementations. In all our studies we have used the GNU Compiler Collection (versions 4.6 and 4.7).
